# Idelalisib impairs TREM-1 mediated neutrophil inflammatory responses

**DOI:** 10.1038/s41598-018-23808-2

**Published:** 2018-04-03

**Authors:** Astrid Alflen, Nicole Stadler, Pamela Aranda Lopez, Daniel Teschner, Matthias Theobald, Georg Heß, Markus P. Radsak

**Affiliations:** Department of Hematology, Medical Oncology, & Pneumology, University Medical Center of the Johannes Gutenberg University, Mainz, Germany

## Abstract

Triggering receptor expressed on myeloid cells (TREM)-1 on polymorphonuclear neutrophils (PMN) regulates innate immune activation in infectious and non-infectious conditions. TREM-1 ligation activates phosphatidyl-inositol 3 kinase (PI3K) triggering all neutrophil effector functions. As idelalisib is a PI3K inhibitor in clinical use for the treatment of non-Hodgkin lymphomas, we asked whether this inhibitor affects PMN functionalities. We analyzed PMNs from healthy donors or lymphoma patients for oxidative burst, phagocytosis, activation markers and IL-8 release upon TREM-1 or TLR ligation *ex vivo*. In addition, we performed western blot analyses to characterize the signaling events inhibited by idelalisib and other PI3K inhibitors. Upon TREM-1 ligation, the oxidative burst, degranulation, L-selectin shedding and cytokine release were all strongly reduced in the presence of idelalisib along impaired phosphorylation of P38, AKT and ERK by western blot analyses. In line with this, PMNs from patients receiving idelalisib also displayed an impaired TREM-1 mediated PMN activation *ex vivo*. In conclusion, PI3K inhibitors might cause a neutropenia-like susceptibility to infections in patients by leading to impaired PMN functionality. This should be considered when evaluating patients for infections treated with such inhibitors in daily clinical routine.

## Introduction

The 3-phosphorylated inositol lipids have an important role as second messengers in many cellular processes. Hence, the generation of 3-phosphoinositides influences multiple signaling pathways modulating a broad spectrum of fundamental cellular activities. Kinases involved in the synthesis of 3-phosphoinositides include multiple isoforms that are divided into three classes, in which the class I phosphoinositide 3-kinases (PI3Ks) are heterodimeric enzymes consisting of a p110 catalytic subunit (p110α, β, δ or γ)^1^. Association of the regulatory subunit with activated receptor complexes at the plasma membrane allows the catalytic subunit to convert phosphatidylinositol-4,5-bisphosphate to phosphatidylinositol-3,4,5-trisphosphate, a lipid second messenger molecule^2^. Idelalisib (GS-1101, CAL-101, 5-fluoro-3-phenyl-2-[(S)-1-(9H-purin-6-ylamino)-propyl]-3H-quinazolin- 4-one) is a potent small-molecule inhibitor that is highly specific for PI3Kδ leading to the abrogation of PI3K/AKT signaling and the induction of apoptosis in lymphoid cell lines and primary patient samples^3^. Since September 2014, idelalisib is approved for the treatment of chronic lymphatic leukemia (CLL) and follicular lymphoma (FL) in Europe. Investigations especially concerning the combination therapy with monoclonal antibodies revealed that idelalisib affects not only lymphatic cells, but also impairs innate immune functions, e.g. natural killer (NK) cell degranulation, neutrophil activation and phagocytosis by macrophages^4^. This suggests that idelalisib may also impair innate immune responses in patients under treatment with this drug, possibly further aggravating the well-known secondary immunodeficiencies in CLL or FL patients.

Neutrophils are a vital part of the innate immune system protecting the host against microbial and fungal pathogens by their ability to rapidly migrate to a site of infection and employing their potent effector mechanisms. Beyond this, they intimately shape the subsequent adaptive immune responses at various levels^5^. For effective functionality and prevention of secondary damage, neutrophil activation is strictly regulated, mainly by surface receptors such as receptors for complement, chemokines or cytokines or receptors directly recognizing pathogens like Toll-like receptors (TLR), (NOD)-like receptors (NLR)^6^. In this context, the triggering receptor expressed on myeloid cells (TREM)-1 is expressed on neutrophils, and monocytes/macrophages and also implicated in immune activation. TREM-1 belongs to the Ig superfamily and has a short cytoplasmic tail lacking signaling motifs. Therefore, TREM-1 is associated with the transmembrane adapter protein DAP12 for activation leading to release of pro-inflammatory chemokines and cytokines, increased surface expression of cell activation markers and degranulation. TREM-1 plays an important role in innate immune response in infectious settings and sepsis^7,8^ but also in non-infectious conditions like critical limb ischemia^9^ and rheumatoid arthritis^10^. Although so far not completely understood, complexes of peptidoglycan recognition protein 1 (PGLYRP1) and bacterially derived peptidoglycan and high mobility group box-1 (HMGB1) have been identified as ligands for TREM-1^11^.

Several studies have been performed to elucidate TREM-1 downstream signaling pathways and regulation of TREM-1 expression. Inhibitor studies disclosed a PI3K-dependent pathway in lipopolysaccharide (LPS)-induced up-regulation of TREM-1 on monocytes, whereas mitogen-activated protein kinases (P38 MAPK and P42/P44 MAPK) play a limited role^12^. In addition, inhibition of PKA, P38 MAPK and PI3K significantly suppressed prostaglandin E2 (PGE2)-induced TREM-1 expression, whereas a MAPKK inhibitor failed to influence TREM-1 expression, suggesting that PGE2-induced TREM-1 expression was mediated via the PKA, PI3K, and P38 MAPK pathways^13^. Anti-TREM-1 mediated respiratory burst was also significantly impaired when PMN were pretreated *in vitro* with a PI3K specific inhibitor^14^. In accordance with that, using pharmacological inhibitors and Western blot analysis Haselmayer *et al*. demonstrated that PI3K, PLC and P38 MAPK are essential for the TREM-1- and TLR4-induced oxidative burst of human PMN^15^.

In the present study, we were interested to assess whether the PI3K inhibitor idelalisib in clinical use for treatment of CLL and FL affects neutrophil activation. As PI3K is involved in TLR as well as TREM-1 mediated neutrophil activation, we analyzed TLR or TREM-1 mediated activation in the absence or presence of idelalisib *in vitro* as well as in blood samples from idelalisib-treated patients after *ex vivo* stimulation. We demonstrate that idelalisib preferentially abrogated the TREM-1 mediated neutrophil activation of the oxidative burst, degranulation, CD62L shedding and interleukine-8 (IL-8) release and suppresses TREM-1 associated signaling events downstream of PI3K, while only the TLR4 mediated oxidative burst was impaired. Importantly, we were able to confirm this idelalisib-induced impaired neutrophil activation in patients under treatment suggesting that these findings are of clinical relevance. This highlights the importance to monitor patients receiving targeted therapies not only for neutrophil counts, but also for functional defects as infections belong to the major adverse events in patients under treatment with idelalisib.

## Materials and Methods

### Materials

Lipopolysaccharides (LPS) from *Salmonella typhimurium* and phorbol myristate acetate (PMA) were obtained from Sigma-Aldrich (Taufkirchen, Germany). The following antibodies were used for analysis by flow cytometry: anti-CD11b (clone: CBRM1/5) PE and anti-CD62L (clone: DREG-56) APC (BioLegend, San Diego, CA, USA), anti-CD66b (clone: 80H3) FITC (Beckman Coulter, Krefeld, Germany). Other antibodies used were anti-TREM-1 clone 6B1, raised by fusion of SP2/0 myeloma cells (from American Type Culture Collection, Manassas, VA) with splenocytes from a BALB/c mouse immunized with a recombinant sTREM-1 fusion protein and screened against TREM-1, and monoclonal mouse IgG1 clone 4C9^16^. Western blot antibodies: anti-β-Actin mouse mAb (clone: AC-15) (Sigma-Aldrich, Taufkirchen, Germany), polyclonal anti-p44/42 MAPK (Erk 1/2) rabbit Ab, polyclonal anti-phospho-Akt (Thr308) Ab, anti-Akt (pan) rabbit mAb (clone: C67E7), anti-phospho-p38 MAPK (T180/Y182) rabbit mAb (clone: 3D7), polyclonal anti-phospho-Btk (Tyr223), polyclonal anti-rabbit IgG HRP-linked Ab, polyclonal anti-p38 MAPK, anti-phospho-p44/42 MAPK (Erk1/2) (Thr202/Tyr204) mouse mAb (clone: E10), polyclonal anti-mouse IgG HRP-linked Ab (all from Cell Signaling Technology, Danvers, MA, USA), polyclonal anti-p-PI 3-kinase pp110 δ (Tyr 485), anti-PI 3-kinase p110 δ (clone: H-219) (Santa Cruz Biotechnology, Dallas, TX, USA), anti-BTK Ab (clone: Y440) (Abcam plc, Cambridge, UK). PI3K inhibitors: CAL-101 (idelalisib, GS-1101), CZC24832, TGX-221, HS-173 (all from Selleckchem, Munich, Germany). For detailed specificity of the inhibitors according to the manufacturer see Table 1.

**Table 1:** PI3K inhibitors.

PI3K inhibitor	product name	α	β	γ	δ
**α**	HS-173	++++			
**β**	TGX-221	+	++++		++
**γ**	CZC24832		+	+++	+
**δ**	CAL-101, idelalisib, GS-1101	+	+	++	++++

Selectivity of the respective inhibitors to various PI3K isoforms (according to the manufacturer Selleckchem).

### Ethics, consent and permissions

All human studies were performed after obtaining informed consent from healthy volunteer donors and patients and were approved by the local ethics committee according to the institutional guidelines (Landesärztekammer Rhineland-Palatine, Approval no. 837.224.10 (7233)).

### PMN purification

Neutrophils were isolated by dextran sedimentation and Histopaque^®^ centrifugation. Briefly, red blood cells were sedimented on dextran solution in a 1:1 ratio for 30 min at room temperature (RT). Afterwards the upper part was layered over the gradient medium (Histopaque^®^) again in a 1:1 ratio and centrifuged at 1700 rpm for 30 min at RT. The lower neutrophil-containing band was harvested and remaining contaminating red blood cells were removed by a hypotonic lysis. When indicated, cells were preincubated at 37 °C for 30 min with respective inhibitors or vehicle (dimethyl sulfoxide, DMSO) before stimulation.

### Cell stimulation

For assessment of the oxidative burst activity, degranulation, L-selectin shedding, IL-8 secretion, viability and Western blot analysis anti-TREM-1 was coated (10 µg/ml) in flat-bottom plates (Greiner Bio One, Frickenhausen, Germany). Cross-linking of anti-TREM-1 was performed with a secondary goat anti-mouse F(ab’)2 (Dianova, Hamburg, Germany) for experiments followed by Ca^2+^ mobilization assay. LPS was applied at 1 µg/ml and PMA 10 nM.

### Detection of oxidative burst

The presence of hydrogen peroxide was detected by oxidation of non-fluorescent dichloro-fluorescein diacetate (DCFH-DA 3.3 nM from Sigma-Aldrich, Taufkirchen, Germany) into green fluorescent dichloro-fluorescein as described previously^16^ and measured as kinetics using a microplate reader (Tecan Infinite, Tecan Group Ltd., Männedorf, Switzerland) immediately after sample preparation and cell activation or by flow cytometry (LSR II, Becton Dickinson, Heidelberg, Germany) after cell activation for one hour at 37 °C. Specific fluorescence index of stimulated cells was obtained by subtraction of the background fluorescence of labeled cells incubated in medium alone at the corresponding time points for kinetics or by calculation of the specific fluorescence index (SFI = mean fluorescence intensity of activated sample/mean fluorescence intensity of unstimulated control) for flow cytometry samples.

### Flow cytometry

Neutrophils were isolated and preincubated as described above, cell activation was permitted for 1 h at 37 °C. Phagocytosis was assessed by simultaneous incubation with Fluoresbrite™ Polychromatic Red 1.0 Micron Microspheres (Polysciences, Warrington, PA, USA) and PE intensity was measured via flow cytometry. For degranulation (elevated CD66b and CD11b surface expression) and L-selectin shedding (reduced CD62L surface expression) samples were washed and stained with respective antibodies. Fluorescence signals were detected via flow cytometry. Phagocytosis is indicated as SFI (see above), degranulation is depicted as index (activated sample/unstimulated control) after gating on CD11b^high^ cells, likewise for CD66b. L-selectin shedding represents the percentage of CD62L^low^ PMN.

### Detection of IL-8 release

Supernatants from stimulated cells were collected after 16 h at 37 °C, frozen at –20 °C until required and analyzed by standard enzyme-linked immunosorbent assay (ELISA) for IL-8 (R&D Systems, Abdingdon, UK) according to the manufacturer’s instruction.

### Viability assay

Cells were activated for 16 h and washed twice. Viability was assessed using CellTiter 96^®^ Aqueous One Solution Cell Proliferation Assay (Promega, Madison, WI, USA). As viability control rhGM-CSF (R&D systems, Abdingdon, UK) 10 ng/ml was used and cyloheximide (Sigma-Aldrich, Taufkirchen, Germany) 100 µg/ml treated samples represented dead cells.

### Calcium mobilization assay

Neutrophils (1 × 10^6^/ml) were loaded with the Ca^2+^-sensitive fluorogenic dye FLUO-3/AM (2 µM; Molecular Probes, Eugene, Oreg., USA) at 37 °C for 30 min and washed twice. Stimuli were added as indicated, fluorescence signals were acquired by flow cytometry and analyzed with FlowJo software V8.7.1 (Tree Star Inc., Ashland, Oreg., USA)^15^. Ionomycin calcium salt from *Streptomyces conglobatus* (1 µM; Sigma-Aldrich, Taufkirchen, Germany) served as positive control.

### Western blot analysis

After PMN activation for 30 min, cells were lysed with modified urea buffer (7 M urea, 2 M thio-urea, 5 mM DTT, 2% CHAPS, 10 mM PMSF, 0.5 mM Na-orthovanadate, 5 mM NaF, complete protease inhibitor cocktail) and protein concentration was quantified according to the method of Bradford. Samples were then subjected to SDS PAGE. After electrophoresis, proteins were transferred from the gel onto a PVDF membrane (Merck Millipore, Billerica, MA, USA) by a semi-dry process. The membranes were probed with appropriate primary and secondary antibodies conjugated with horseradish peroxidase as indicated and visualized by the ECL detection system as directed by the manufacturer (Pierce, Bonn, Germany).

### Statistical analyses

All graphing and statistical analyses were performed using GraphPad Prism V5.0a (Graphpad, San Diego, CA, USA). For all analyses, a value of p < 0.05 was considered significant. For multiple (group) comparisons, one- or two-way ANOVA and Bonferroni’s post-test were used.

## Results

### Idelalisib impairs neutrophil functions *in vitro*

**Figure 1:**
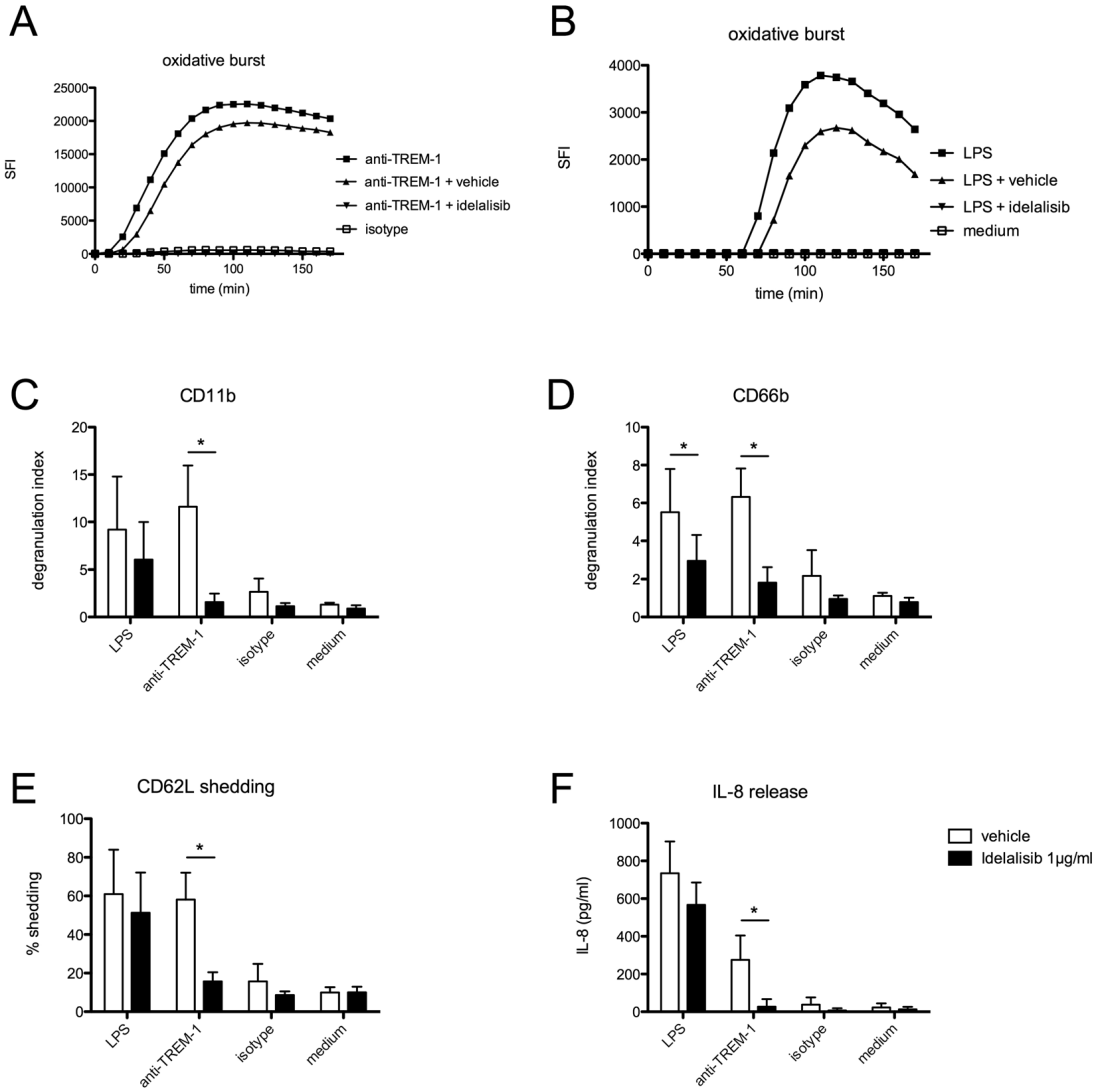
Idelalisib impairs human neutrophil functions *in vitro*. PMN from healthy human donors were isolated and preincubated with idelalisib (1 µg/ml) or vehicle (DMSO) for 30 min at 37 °C. Cells were activated with indicated stimulus (anti-TREM-1 mAb, isotype matched control mAb, LPS at 1 µg/ml) and neutrophil activation was detected as oxidative burst kinetics (**A,B**) for 3 h. One out of five experiments is depicted. Further samples were activated for 1 h at 37 °C and analyzed by flow cytometry. Degranulation was detected as enhanced surface expression of (**C**) CD11b (n = 5) and (**D**) CD66b (n = 5) surface expression and (**E**) CD62L shedding (n = 4). For IL-8 release cells were incubated with indicated stimuli for 16 h and (**G**) IL-8 was measured out of cell supernatants by standard ELISA (n = 3). (*) indicates a significant difference (p < 0.05) by two-way ANOVA with Bonferroni’s posttest. Mean plus SD is depicted.

To assess whether idelalisib affects TREM-1 and TLR4 mediated neutrophil activation, we stimulated neutrophils from healthy volunteer donors with TREM-1 specific mAb or LPS in the absence or presence of idelalisib 1 µg/ml. The concentrations were chosen according to the blood plasma concentrations of patients receiving the standard treatment of idelalisib 150 mg twice per day^17^. As positive control for neutrophil activation and to exclude toxic effects, we included stimulations with PMA as irreversible activator of protein kinase C showing an unimpaired oxidative burst activity in the presence of idelalisib (not shown). In contrast to this, the TREM-1 induced oxidative burst was completely abolished in the presence of idelalisib, while this was not the case with the vehicle (DMSO) control (Fig. 1A). Interestingly, the TLR4 induced oxidative burst (mediated by LPS) was also suppressed by idelalisib (Fig. 1B).

To confirm this impairment of neutrophil functionality, we extended our analyses to further neutrophil functions: While TREM-1 or LPS induced phagocytosis or cell viability were only mildly impaired by idelalisib (not shown), degranulation (CD11b and CD66b) (Fig. 1C,D) as well as CD62L shedding (CD62L) (Fig. 1E) upon TREM-1 ligation were completely abrogated after pretreatment with idelalisib. Furthermore, TREM-1 mediated IL-8 release was abolished in idelalisib-pretreated neutrophils (Fig. 1F). In contrast, LPS triggered neutrophil activation displayed only mild impairment by idelalisib not reaching significance (Fig. 1F). Of note, LPS induced prolonged neutrophil survival was not impaired by idelalisib (not shown) indicating that the activation of PI3K is dispensible in this context. Supplementary Table 1 shows the range of individual data points.

Taken together, idelalisib abrogates TREM-1 mediated neutrophil activation in terms of the oxidative burst, degranulation and IL-8 release, while TLR4 mediated neutrophil activation is only affected concerning the oxidative burst, suggesting that this drug may be useful in modulating neutrophil functionality in a targeted way.

### Idelalisib impairs TREM-1 signaling in neutrophils *in vitro*

**Figure 2:**
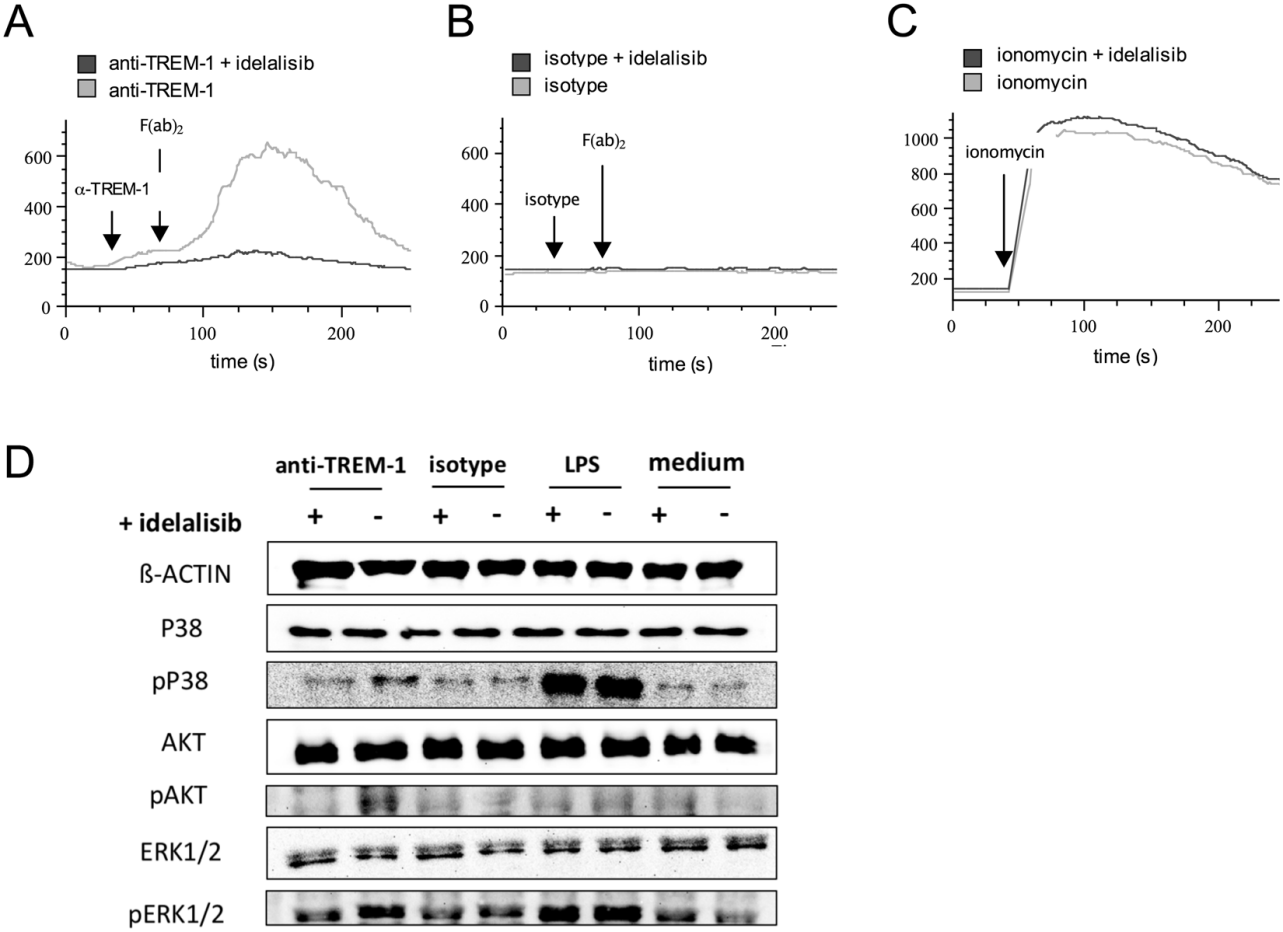
Idelalisib impairs calcium flux and TREM-1 signaling in neutrophils *in vitro*. PMN from healthy human donors were isolated, preincubated with idelalisib 1 µg/ml and loaded with FLUO-3/AM (both at 37 °C for 30 min) for calcium flux assay. (**A**–**C**) Fluorescence signals were detected by flow cytometry for 30 s. For PMN activation indicated stimuli ((**A**) anti-TREM-1, (**B**) isotype matched control mAb, (**C**) ionomycin) was added. For cell stimulation via TREM-1 receptor (and isotype control) cross-linking was performed with a secondary antibody again after 30 s; fluorescence signals were gained for further 5 min. Ionomycin served as positive control. One out of three experiments is depicted. (**D**) For protein analysis PMN were activated with indicated stimuli (anti-TREM-1 antibody, matched control mAb, LPS) for 30 min after preincubated with idelalisib as described above. Proteins were extracted with an urea-based lysis buffer, SDS PAGE was performed and proteins were blotted by a semi-dry process. Protein activation was analyzed by staining of phosphorylated and non-phosphorylated proteins. ß-actin served as loading control. One out of three experiments is depicted.

Calcium flux in neutrophils marks an important step in neutrophil activation and can be triggered by TREM-1 ligation^7^. Hence, we analyzed intracellular Ca^2+^ levels in PMN loaded with the Ca^2+^-sensitive fluorogenic dye FLUO3-AM upon TREM-1, TLR4 ligation and ionomycin as positive control. We found that soluble activation of isolated PMN with anti-TREM-1 antibody and crosslinking with F(ab’)2 antibody induced a transient increase in intracellular Ca^2+^ levels (Fig. 2A) as described previously^7^. This was abolished in the presence of idelalisib (Fig. 2A). In contrast, in the presence of an isotype matched control mAb (Fig. 2B) and TLR4 ligation (data not shown), we were unable to observe Ca^2+^ flow which is in line with previous data^18^. Positive control with Ca^2+^ ionophore ionomycin was not impaired by the presence of idelalisib (Fig. 2C). These results indicate that Ca^2+^ flux triggered by TREM-1 occurs downstream of PI3K and is inhibited by idelalisib.

To further resolve how the downstream signaling events after TLR4 or TREM-1 ligation are affected by idelalisib, we examined the phosphorylation of involved molecules in the presence of this inhibitor by Western blot. As expected, neutrophils steadily expressed P38 MAPK, AKT, ERK1/2 (Fig. 2D), PI3Kδ and BTK (not shown). After ligation of TREM-1, phosphorylation of P38, ERK1/2 (Fig. 2D) and BTK (not shown) were induced. We found that phosphorylation and thereby activation of these proteins were highly impaired in the presence of idelalisib after PMN stimulation by TREM-1 ligation (Fig. 2D). In contrast, LPS mediated phosphorylation of these proteins remained unchanged (Fig. 2D). Interestingly, AKT phosphorylation was only detected after TREM-1 ligation and the presence of idelalisib strongly inhibited the phosphorylation of AKT. Whole Western blots and quantitative analysis are shown in Supplementary Figure 1 and 2.

These results indicate that the PI3K inhibitor idelalisib suppresses P38 MAPK, ERK1/2 and AKT activation placing these signaling events downstream of PI3K in the TREM-1 signaling cascade. In contrast to this and mostly in line with our previous results on the biological neutrophil activity, idelalisib does not significantly affect TLR4 signaling in neutrophils, at least in terms of P38, AKT and ERK1/2 activation.

### Impaired TREM-1 mediated neutrophil activation is not PI3Kδ specific

**Figure 3:**
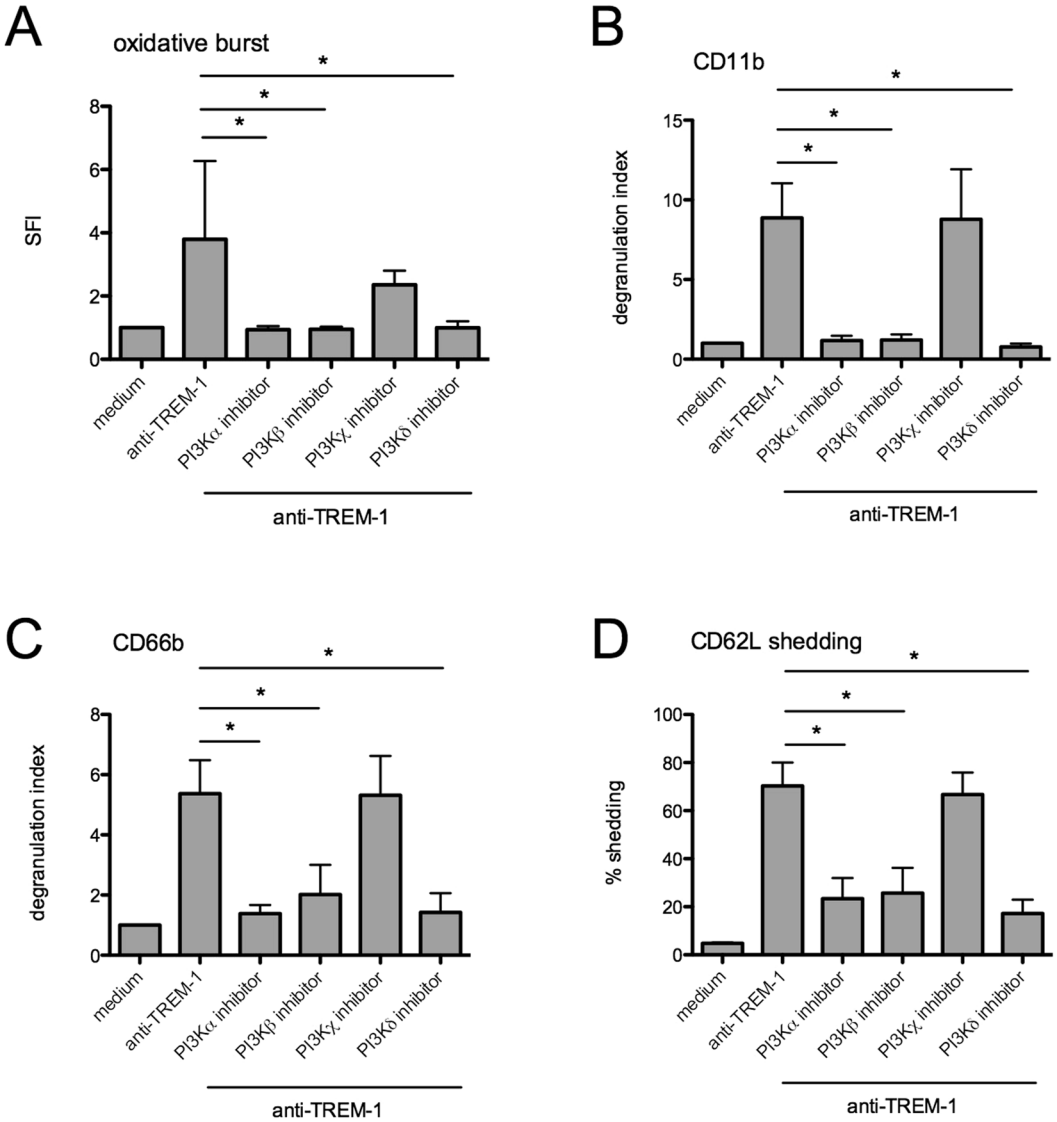
Impaired TREM-1 activation in neutrophils is not specific to PI3Kδ. PMN from healthy human donors were isolated and preincubated with respective PI3K inhibitors 1 µg/ml for 30 min at 37 °C. Cells were activated with anti-TREM-1 antibody and (**A**) oxidative burst was assessed via fluorescence kinetic. Depicted is the SFI after 2 h (n = 5). Further samples were stimulated for 1 h and neutrophil activation was detected as degranulation: (**B**) CD11b (n = 5) and (**C**) CD66b (n = 5) surface expression and (**D**) CD62L shedding (n = 5) by flow cytometry. (*) indicates a significant difference (p < 0.05) by one-way ANOVA with Bonferroni’s posttest. Mean plus SD is depicted.

We observed significantly impaired neutrophil functions after TREM-1 ligation upon idelalisib pretreatment. Nevertheless, our results did not address so far whether these effects are specifically related to PI3Kδ and whether this is the central isoform involved in TREM-1 signaling. Therefore, we examined the impact of various isoform specific PI3K inhibitors on neutrophil activation. Isolated human PMN were again preincubated with the respective inhibitors as indicated and activated by anti-TREM-1. The oxidative burst activity was abolished after pretreatment of cells with inhibitors specific to PI3Kα, -β and -δ isoforms, while a PI3Kγ inhibitor only mildly influenced the generation of reactive oxygen species (Fig. 3A). TREM-1 mediated phagocytosis was not significantly altered (not shown), whereas degranulation (CD11b and CD66b) as well as CD62L shedding (CD62L) were highly impaired after pretreatment of neutrophils with PI3Kα, -β and -δ inhibitors (Fig. 3B–D). IL-8 release showed a similar pattern, although without statistical significance (not shown). Supplementary Table 2 shows the range of individual data points.

Collectively, the results indicate that the PI3Kα, -β and -δ isoforms are crucial for the initiation of neutrophil effector functions, while PI3Kγ is apparently not involved.

### Impaired neutrophil functions in idelalisib-treated patients after *ex vivo* activation

**Figure 4:**
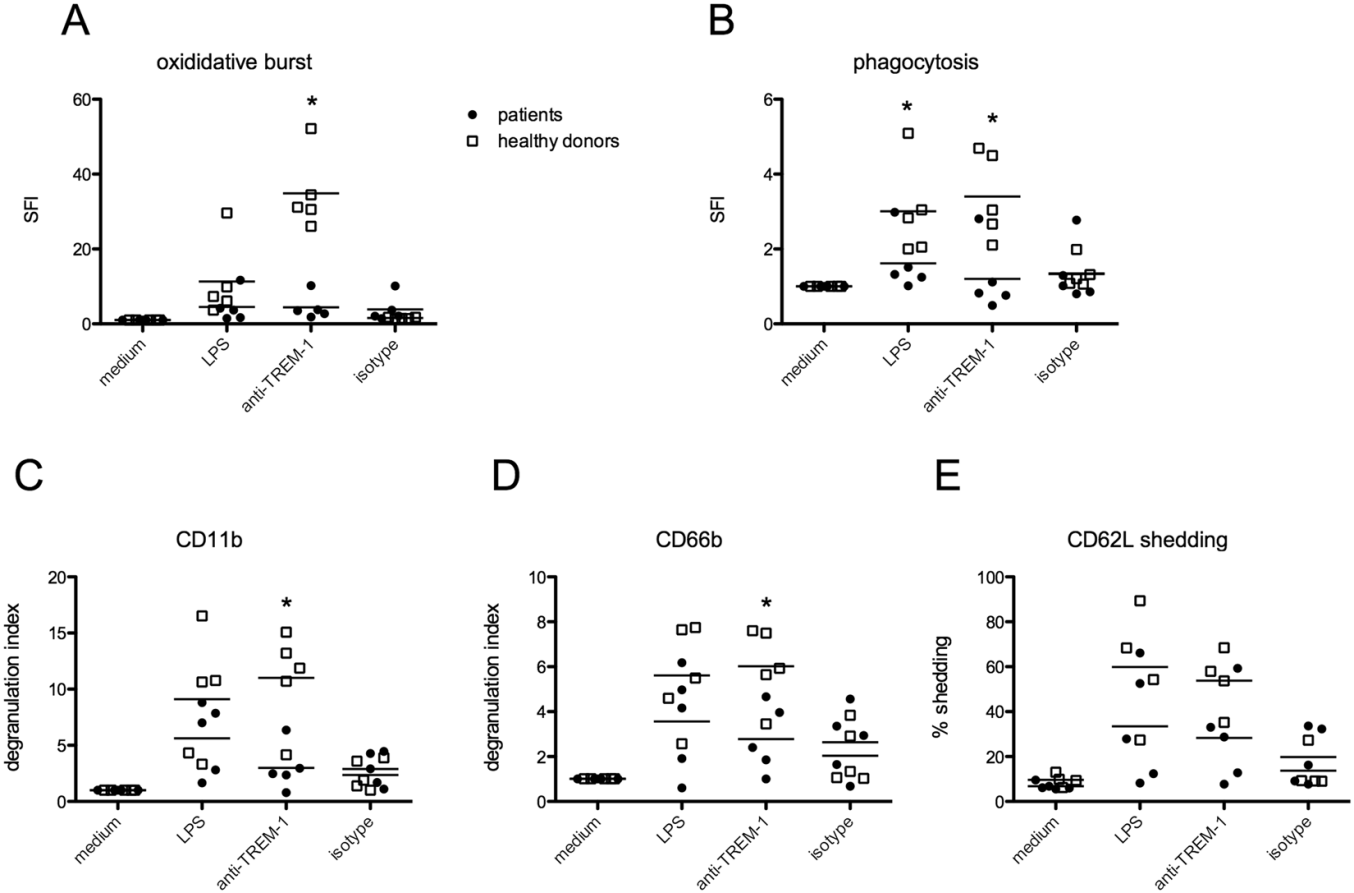
Impaired neutrophil functions in idelalisib-treated patients after *ex vivo* activation. PMN from idelalisib-treated patients (n = 5) and healthy human donors (n = 5) were isolated. Cells were activated as indicated (anti-TREM-1 antibody, matched control mAb, LPS) for 1 h at 37 °C and neutrophil functions were assessed via flow cytometry. Mean and SD of (**A**) oxidative burst, (**B**) phagocytosis, degranulation – (**C**) CD11b and (**D**) CD66b surface expression and (**E**) CD62L shedding are depicted. (*) indicates a significant difference (p < 0.05) by two-way ANOVA with Bonferroni’s posttest.

To assess whether the impaired neutrophil functionality in the presence of idelalisib may be also relevant *in vivo*, we finally analyzed neutrophil functions *ex vivo* from five patients suffering from B-cell non-Hodgkin lymphomas receiving idelalisib treatment. Patients had no obvious signs of infection at presentation and day of blood donation. The detailed patient characteristics are summarized in Table 2. Interestingly, neutrophils from idelalisib-treated patients showed highly impaired oxidative burst after TREM-1 ligation compared to healthy donors (Fig. 4A). Furthermore, phagocytosis was impaired after TREM-1 as well as after LPS activation of isolated neutrophils *ex vivo* (Fig. 4B). While TREM-1 mediated degranulation (CD11b and CD66b) was significantly reduced (Fig. 4C,D) CD62L shedding was additionally impaired when PMN activation was triggered by TLR4 ligation (Fig. 4E).

**Table 2:** Patient characteristics.

diagnosis	sex	age	pretreatments	dosage idelalisib	WBC (/nl) PMN (%)	CRP
chronic lymphatic leukemia (B-CLL)	m	70	rituximab/bendamustin (C2) ibrutinib (8 month)	150 mg 1-0-1	3.9649	4.0
B-prolymphocytic leukemia (B-PLL)	m	70	R-CHOP (C8) autologous PBSCT FCR (C5) rituximab/bendamustin (C6) radiotherapy (30 Gy) rituximab/chlorambucil (C3) ofatumomab-COP (C12)	150 mg 1-0-1	5.0968	17
B-CLL	m	67	none	100 mg 1-0-1	24.77	12
follicular lymphoma (FL)	w	46	rituximab/bendamustin (C6) R-CHOP (C6) R-DHAP (C2) R-BEAM and autologous PBSCT idelalisib (7 month) allogenic PBSCT (HLA identical) donor lymphocyte infusion idelalisib (6 month)	150 mg 1-0-1	6.3169.4	6.2
B-CLL	m	82	n.a.	n.a.	n.a.	n.a.

WBC = White blood cells. CRP = C-reactive protein. R-CHOP = rituximab, cyclophosphamide, doxorubicin, vincristine, prednisone. PBSCT = peripheral blood stem cell transplantation. FCR = fludarabin, cyclophosphamide, rituximab. DHAP = dexamethasone, high-dose cytarabine, cisplatin. BEAM = carmustin, etoposide, cytarabine, melphalan. C = cycles of chemotherapy.

**Figure 5:**
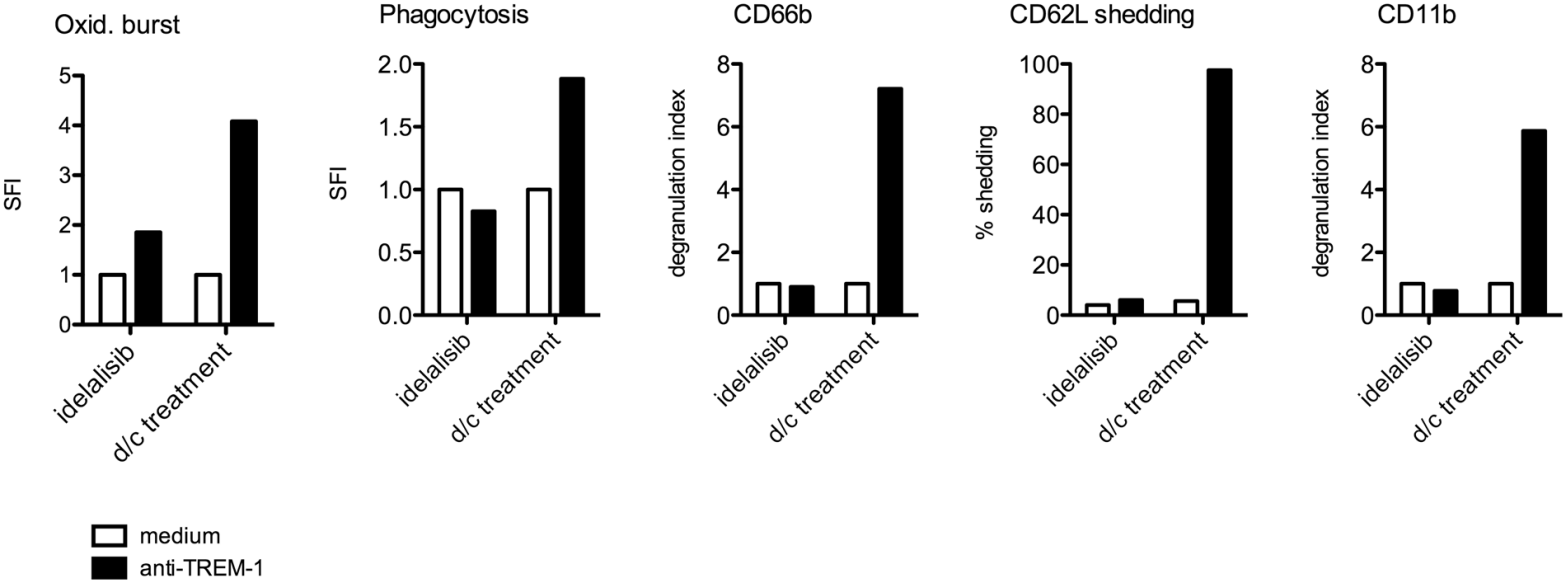
Neutrophil functions are restored after discontinuation of idelalisib treatment. PMN from one patient during idelalisib therapy and after discontinuation (d/c) of treatment were isolated. Cells were activated with anti-TREM-1 antibody for 1 h at 37 °C and neutrophil functions were assessed via flow cytometry. Mean and SD of oxidative burst, phagocytosis, degranulation (CD11b and CD66b) and CD62L shedding are depicted.

Beyond this, we were able to examine neutrophil functions from a patient during idelalisib treatment and several months after discontinuation of the therapy due to stable disease. Interestingly while neutrophil functions were highly impaired during idelalisib intake and *ex vivo* activation via TREM-1, neutrophils showed normal activation profiles after discontinuation of this treatment (Fig. 5).

These data indicate that idelalisib impairs neutrophil functionality in response to TREM-1 ligation and in addition also in response to TLR4 ligation. The latter we have not been able to detect in this way in our *in vitro* texperiments. However, this suggests that PI3K inhibition using idelalisib affects neutrophil functionality in a complex and clinically relevant way, as the reduced neutrophil reactivity in response to TLR4 ligation was only detectable *in vivo*.

## Discussion

Neutrophils are the major pathogen-fighting immune cells in organisms. Central to their function is their ability to be recruited to sites of infection, to recognize and phagocyte microbes and to kill pathogens through a combination of cytotoxic mechanisms. These include the production of reactive oxygen species, the release of antimicrobial peptides, and the expulsion of their nuclear contents to form neutrophil extracellular traps^19^. The critical role and necessity of these cells is especially obvious in hematologic and oncologic patients suffering from neutropenia due to chemotherapy where febrile neutropenia is one of the most frequent and serious complications. A prompt identification of infection and empirical antibiotic therapy can prolong survival. Therefore, a risk assessment, management of infections and prophylaxis are absolutely essential^20^. In these group of patients infections encountered are usually due to alterations in immune function as a consequence of the basic disease, the therapy for the disease, or both in combination^21^. While neutropenia frequently occur due to drug treatment, it can also be based on a disease by itself (e.g. cyclic neutropenia), whereas functional defects of neutrophil are rare, usually associated with an underlying disease (e.g. chronic granulomatous disease)^22,23^ and are not tested in daily clinic routine. Besides the advent of targeted therapies affecting specific kinases in malignant diseases, therapy-associated infectious complications occur as well. Hereby the severity depends on the specific pathway causing targeted or off-target activity of the kinase inhibitor. As pointed out for the treatment of inhibitors of the B-cell receptor (BCR) signal cascade, ibrutinib and idelalisib intake is highly associated with infectious complications, in which respiratory infections such as pneumonia are one of the typical manifestation, reviewed by Reinwald *et al*.^24^. In the initial phase 1 study of idelalisib for CLL patients, serious adverse events were frequently present with ≥ grade 3 pneumonia occurring in 20% and neutropenic fever in 11%^25^. Later, side effects like severe and recurrent infections were announced in a “Dear Doctor Letter (ger. Rote-Hand-Brief)” (1101-16-208) by the German Federal Institute for Drugs and Medical Devices, recommending limited clinical use of this drug. Further clinical observations of infectious complication and experimental data suggest that this approved specific PI3Kδ inhibitor impairs innate immunity^4^. This was also recently highlighted regarding the BTK inhibitor ibrutinib and TREM-1 mediated neutrophil activation, indicating that targeted therapies do not cause impaired neutrophil counts but alter neutrophil functions^26^. TREM-1 mediates activation of neutrophils and monocytes^7^ and contributes to the amplification of the innate inflammatory responses in severe infection and sepsis^8,27^. While it has been shown that PI3K plays an important role in TREM-1 signaling^14,15^, the isoform is unknown so far. In this study we shed light on the aspect of impaired neutrophil functions caused by the specific PI3Kδ inhibitor idelalisib. Here we showed, that *in vitro* treatment of healthy human PMN with idelalisib prior to activation by anti-TREM-1 antibody highly impairs anti-infective functions of these cells (Fig. 1) as well as TREM-1 mediated signaling (Fig. 2). To elucidate whether PI3Kδ is the PI3K isoform involved in TREM-1 signaling or if this is a general inhibitory effect we used different specific PI3K inhibitors in the same experimental setup. We found that PI3Kα, -β and -δ inhibitors show similar profiles of PMN impairment, while a PI3Kγ inhibitor had no impact on neutrophil activation (Fig. 3). PI3Ks are divided into three classes based on their structures and substrate specificities and class I PI3Ks are further divided into subclasses IA and IB based on their modes of regulation. Class IA PI3Ks are heterodimers that contain a p110 catalytic subunit and a p85 regulatory subunit including the three highly homologous class IA catalytic isoforms p110α, p110β and p110δ. Class IB PI3Ks are heterodimers of a p110γ catalytic subunit coupled with the regulatory isoforms p101 or p87^28^. Therefore, the observed results are most likely unspecific effects of PI3K class IA inhibition.

To elucidate if idelalisib alters PMN functions *in vivo* we analyzed blood samples from patients receiving this drug and compared PMN functions to those of healthy controls after *ex vivo* activation. Indeed, neutrophils from idelalisib-treated patients showed an impaired activation profile due to TREM-1 and partly TLR4 ligation (Fig. 4). However, our analysis was focused on activation of PMN due to specific stimuli like LPS and anti-TREM-1 compared to non-activated (medium control) cells. This might also be due to a preactivated state of PMN that probably does not allow further activation to a specific stimulus, no matter whether via TREM-1 or TLR4. This again could be due to the treatment, but co-founding factors like the disease itself cannot be excluded. To shed more light on that, we examined blood samples from a patient during idelalisib therapy and after discontinuation of treatment. While neutrophils of this patient revealed impaired functions to specific stimuli during idelalisib treatment, PMN functions were restored when therapy was terminated (Fig. 5). However, the disease was at a stable state at that timepoint, so this question remains unanswered.

Along with these differential effects of idelalisib on TREM-1 and TLR4 mediated neutrophil activation *in vitro* and *in vivo*, signal transduction of this two activating receptor types showed distinct phosphorylation patterns. While phosphorylation of respective proteins was exclusively abrogated in idelalisib-pretreated and TREM-1-activated neutrophils, AKT was exclusively phosphorylated after TREM-1 ligation (Fig. 2). PI3K has been described for playing a role in TREM-1 and TLR4 signaling and is additionally involved in an interaction between TREM-1 and pattern recognition receptors as reviewed by Arts and coworkers^29^. However, activation via TLR4 could also be observed independent of PI3K since PMN functions *in vitro* as well as phosphorylation of downstream proteins are only mildly impaired by idelalisib (Figs 1 and 2). Furthermore controversial data exist for the optimal time point for detecting phosphorylated proteins in these two pathways^15,30^ providing an explanation for the lack of AKT phosphorylation due to TLR4 activation when analyzing proteins only at one time point.

Taken together, our data highlight the property of harmed neutrophil functions in the setting of normal neutrophil counts using targeted therapies. Since patients suffering from neutropenia require special attention by their treating physicians to prevent febrile neutropenia and sepsis^31^, the aspect of impaired neutrophil functions, but normal cell counts are largely not considered in the daily clinical routine so far. Indeed, one aspect of improving medical care for this patients by monitoring neutrophil functions is to first identify relevant activating receptors.

## Electronic supplementary material

Supplementary Information
