# Supplementary material for: Idelalisib impairs TREM-1 mediated neutrophil inflammatory responses
Source: Sci Rep. 2018 Apr 3;8:5558. doi: 10.1038/s41598-018-23808-2 (PMC5882939; doi:10.1038/s41598-018-23808-2)
Supplement: Supplementary file 1 — Supplementary Information [file 41598_2018_23808_MOESM1_ESM.pdf]

Supplementary Data

to

Idelalisib impairs TREM-1 mediated neutrophil inflammatory responses

by

Astrid Alflen, Nicole Stadler, Pamela Aranda Lopez, Daniel Teschner, Matthias Theobald, Georg Heß, Markus P. Radsak

Supplementary Table 1

This table gives an overview on the range of the individual data points of the bar graphs.

**CD11b**		
	vehicle	*idelalisib 1µg/ml*
LPS	2.9-16.9	1.9-11.8
anti-TREM-1	4.4-16.2	0.8-3.0
isotype	1.3-4.5	0.6-1.4
medium	1.2-1.7	0.5-1.4
**CD66b**		
	vehicle	*idelalisib 1µg/ml*
LPS	2.2-7.6	1.2-4.5
anti-TREM-1	3.8-7.8	0.8-2.7
isotype	1.0-3.0	0.8-1.3
medium	0.9-1.4	0.8-1.1
**CD62L**		
	vehicle	*idelalisib 1µg/ml*
LPS	27.1-86.3	27.5-80.6
anti-TREM-1	36.5-71.1	9.7-22.3
isotype	8.5-28.2	5.6-9.8
medium	5.7-12.2	5.8-12.8
**IL-8 release**		
	vehicle	*idelalisib 1µg/ml*
LPS	558-892	438-670
anti-TREM-1	178-421	0-74
isotype	15-82	0-20
medium	0-44	0-26

Supplementary Table 2

This table gives an overview on the range of the individual data points of the bar graphs.

	**oxidative burst**	**CD11b**
medium	1-1	1-1
anti-TREM-1 (w/o inhibitor)	1.8-8.1	6.7-12.0
PI3Kα inhibitor	0.8-1.1	0.8-1.6
PI3Kβ inhibitor	0.9-1.0	0.7-1.5
PI3Kγ inhibitor	1.6-2.7	6.1-13.8
PI3Kδ inhibitor	0.8-1.3	0.5-1.1
	**CD66b**	**CD62L**
medium	1-1	4.3-5.2
anti-TREM-1 (w/o inhibitor)	4.6-6.9	56.5-81.3
PI3Kα inhibitor	1.1-1.7	12.0-33.1
PI3Kβ inhibitor	0.8-3.3	17.7-40.4
PI3Kγ inhibitor	4.3-7.3	54.8-78.7
PI3Kδ inhibitor	0.9-2.2	12.2-23.7

A

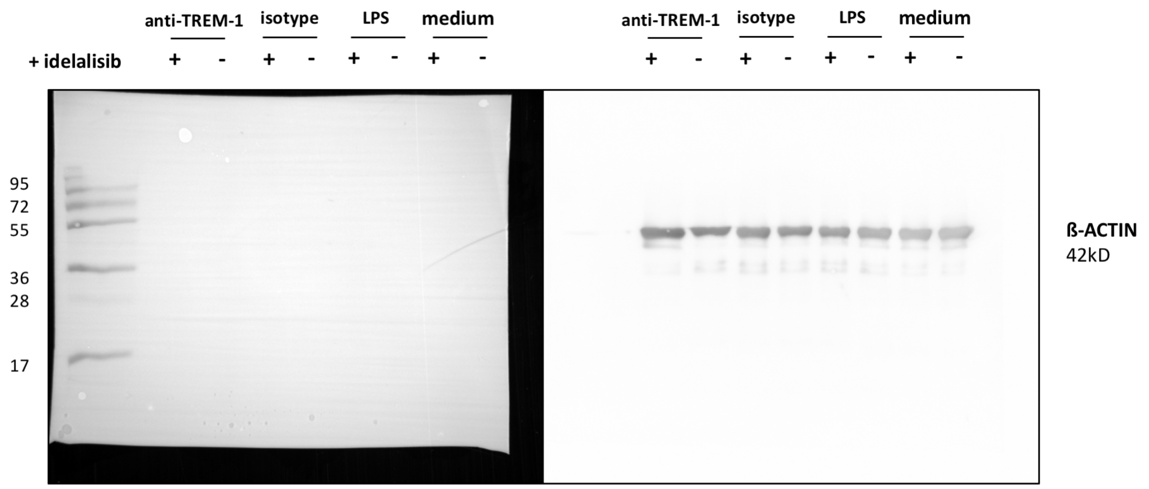
anti-TREM-1
isotype
LPS
medium
+ idelalisib
+ - + - + - + -
95
72
55
36
28
17
anti-TREM-1
isotype
LPS
medium
+ - + - + - + -
ß-ACTIN
42kD

B

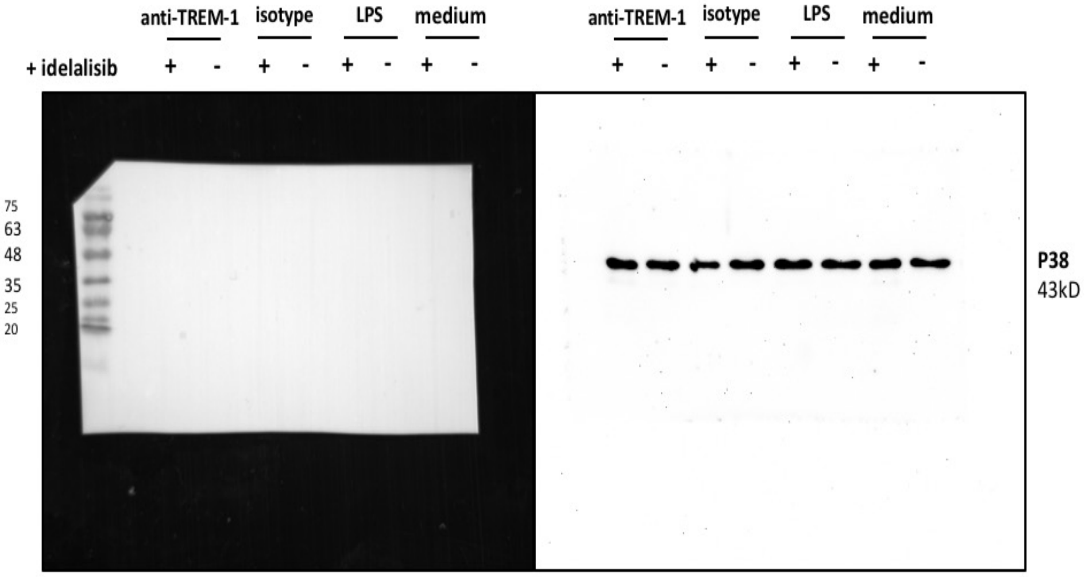
anti-TREM-1
isotype
LPS
medium
+ idelalisib
+ - + - + - + -
75
63
48
35
25
20
anti-TREM-1
isotype
LPS
medium
+ - + - + - + -
P38
43kD

C

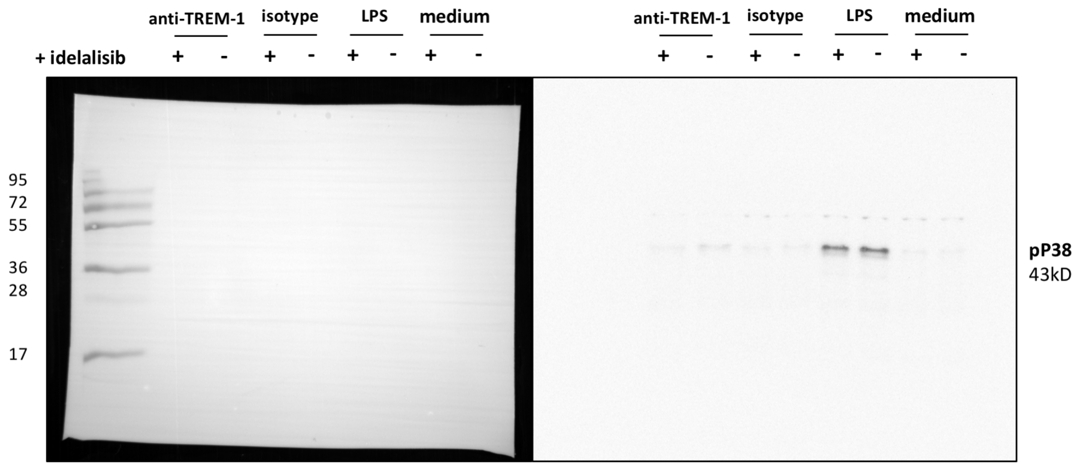
anti-TREM-1
isotype
LPS
medium
+ idelalisib
+ - + - + - + -
95
72
55
36
28
17
anti-TREM-1
isotype
LPS
medium
+ - + - + - + -
pP38
43kD

D

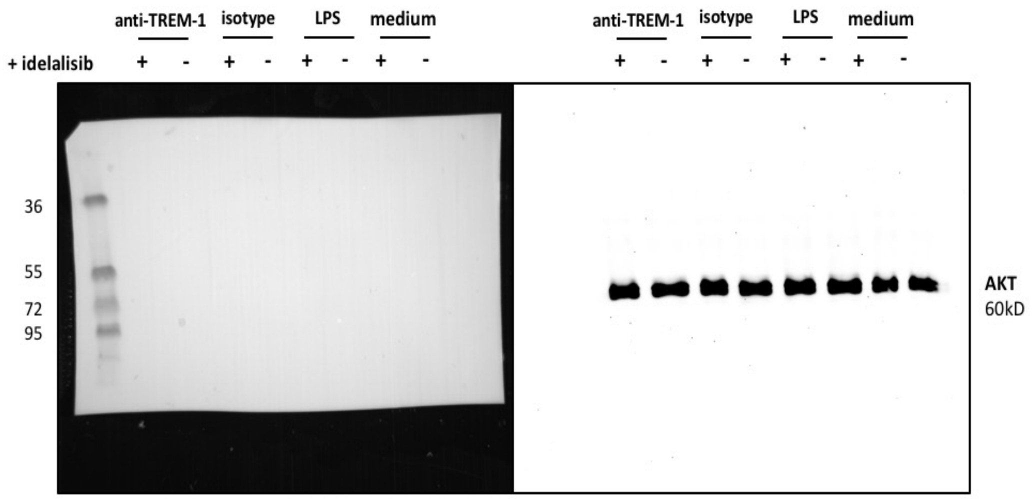
anti-TREM-1
isotype
LPS
medium
+ idelalisib
+ - + - + - + -
36
55
72
95
anti-TREM-1
isotype
LPS
medium
+ - + - + - + -
AKT
60kD

E

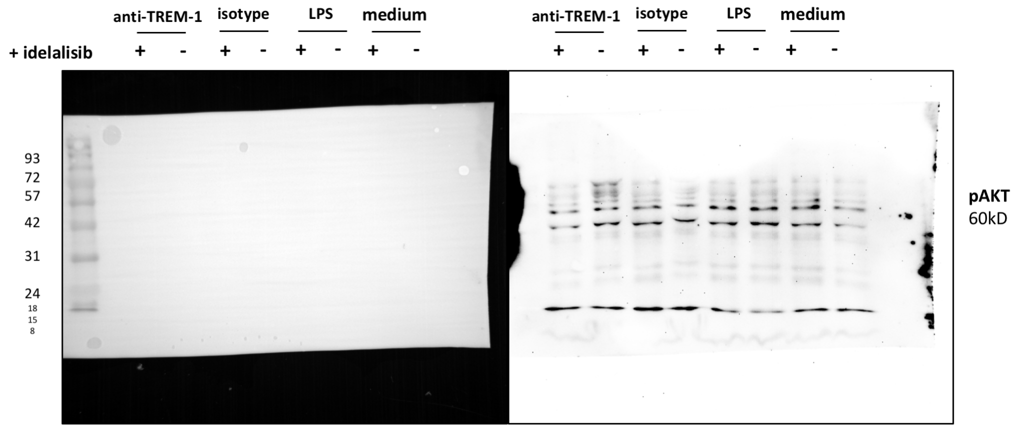
anti-TREM-1
isotype
LPS
medium
+ idelalisib
+ - + - + - + -
93
72
57
42
31
24
18
15
8
anti-TREM-1
isotype
LPS
medium
+ - + - + - + -
pAKT
60kD

F

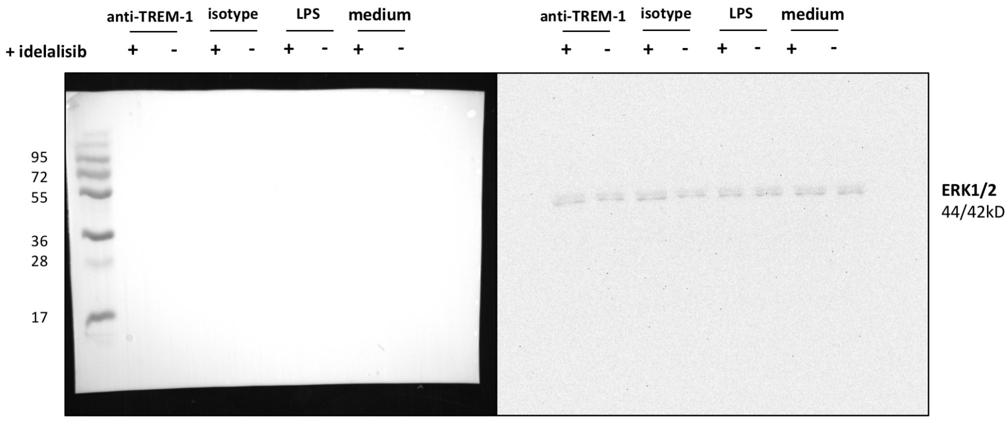
anti-TREM-1
isotype
LPS
medium
+ idelalisib
+ - + - + - + -
95
72
55
36
28
17
anti-TREM-1
isotype
LPS
medium
+ - + - + - + -
ERK1/2
44/42kD

G

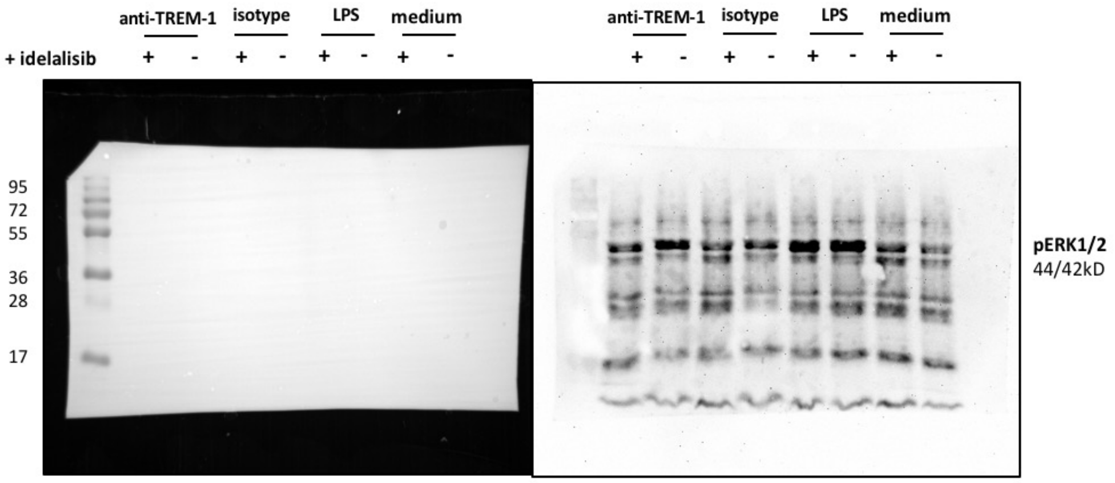

Suppl. Figure 1

Original western blots including protein ladders of data depicted in Figure 2. SDS PAGE was performed and proteins were transferred onto a PVDF membrane by a semi-dry process. Membranes were probed with appropriate primary and secondary antibodies (for respective antibodies see “Materials”) conjugated with horseradish peroxidase and visualized by the ECL detection system. (A) ß-ACTIN, (B) P38, (C) pP38, (D) AKT, (E) pAKT, (F) ERK1/2 and (G) pERK1/2.

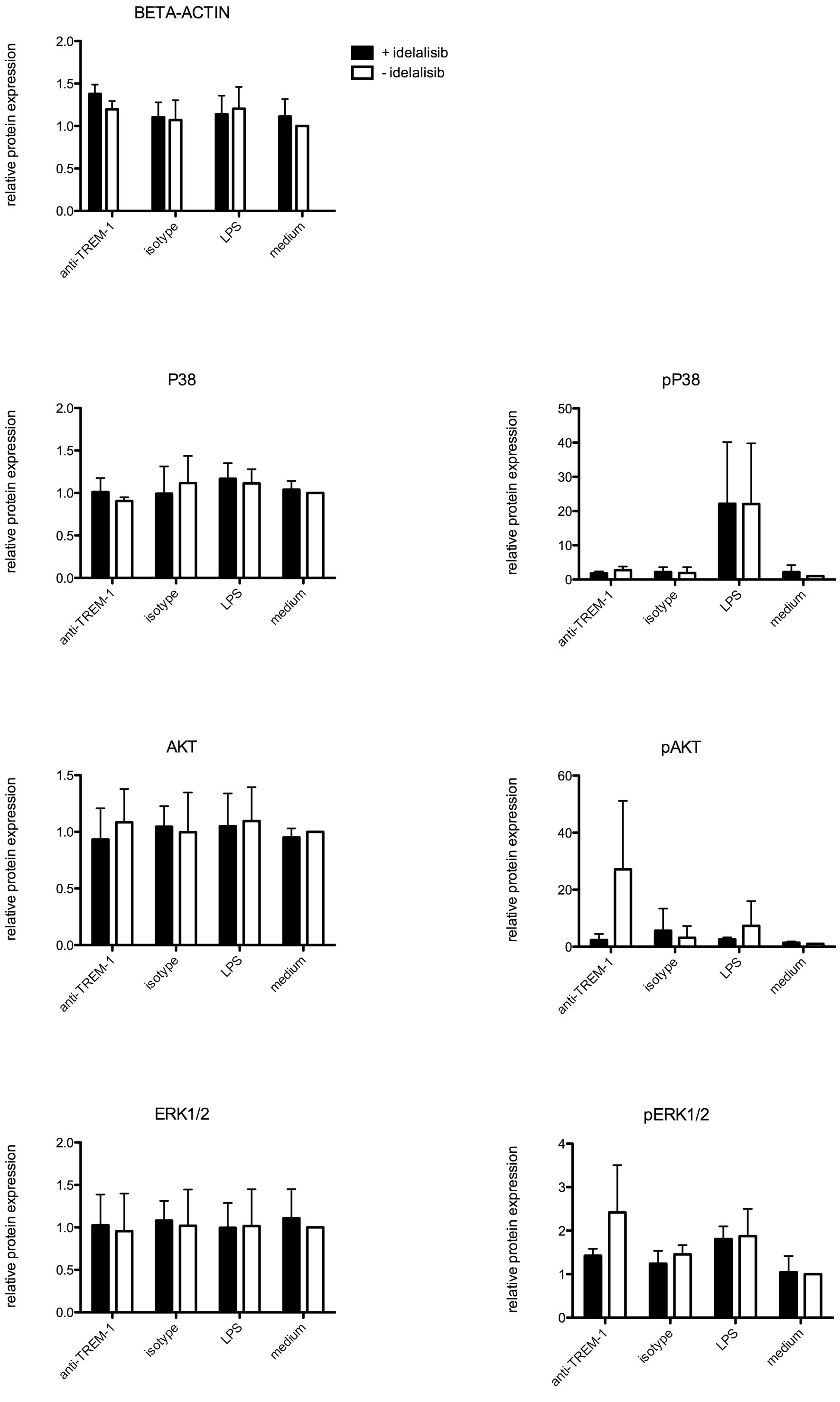

Supplementary Figure 2

Quantitative Western blot results

Western blots were repeated in three experiments and quantitative analysis was performed using Image J software and values were calculated as "relative protein expression" in relation to untreated samples (medium, - idelalisib).
